# Chondroblastic osteosarcoma

**DOI:** 10.4322/acr.2023.466

**Published:** 2024-01-08

**Authors:** Neha Bhardwaj, Vikas Bachhal, Uma Nahar Saikia

**Affiliations:** 1 Post Graduate Institute of Medical Education and Research, Department of Histopathology, Chandigarh, India; 2 Post Graduate Institute of Medical Education and Research, Department of Orthopaedics, Chandigarh, India

**Keywords:** Bone Neoplasms, Femur, Osteosarcoma

Osteosarcoma is an intramedullary high-grade osteoid-producing sarcoma. It has a preference for sites of the most proliferative growth plates.^1^ They occur in the metaphysis of long bones, commonly with direct extension into the adjacent epiphysis and diaphysis. It has a bimodal presentation, with most cases between the ages of 14 and 18 years and a second smaller peak in older adults.^2^ The gross cut surface depends on the type and degree of mineralization of the predominant matrix, which may be dense, solid, tan-white, resembling cortical bone or grey, rubbery or mucoid in the case of cartilaginous component.^3^ Histomorphological diagnosis of osteosarcoma warrants the demonstration of neoplastic bone formation, which may be in any amount to render the diagnosis. Characteristically, fine or coarse lace-like patterns or broad, large sheets of compact bone formed by coalescing trabeculae are noted. The tumor grows in a permeative pattern, replacing the marrow spaces, encasing and eroding the bony trabeculae. The tumor cells generally show marked pleomorphism with abundant mitosis. Based on the predominant matrix, osteosarcomas are subdivided into osteoblastic (76-80%), chondroblastic (10-13%), and fibroblastic (10%) types. In chondroblastic osteosarcoma, the predominant component is hyaline cartilage with severe cytological atypia. The neoplastic cartilage merges with malignant osteoid and shows condensation and spindling of tumor cells at the periphery of these chondroid nodules.^3,4^ This feature is beneficial in small biopsy diagnosis, where it needs to be differentiated from chondrosarcoma. The staging is based on the greatest tumor dimension (≤ 8 cm: T1; > 8 cm: T2) and discontinuous involvement of the primary bone site (T3). Pulmonary, followed by skeletal metastasis, are the most frequent sites of systemic disease.^3^ High-grade osteosarcoma is, therefore, usually treated with preoperative and postoperative chemotherapy, and local control is achieved via surgical resection with wide margins using limb-salvage techniques. Radiation can be used for unresectable tumors.^5^ The histological response to neoadjuvant chemotherapy remains one of the most important prognosticators of overall and disease-free survival, with ≥ 90% necrosis defined as a good response.^6^

**Figure 1:**
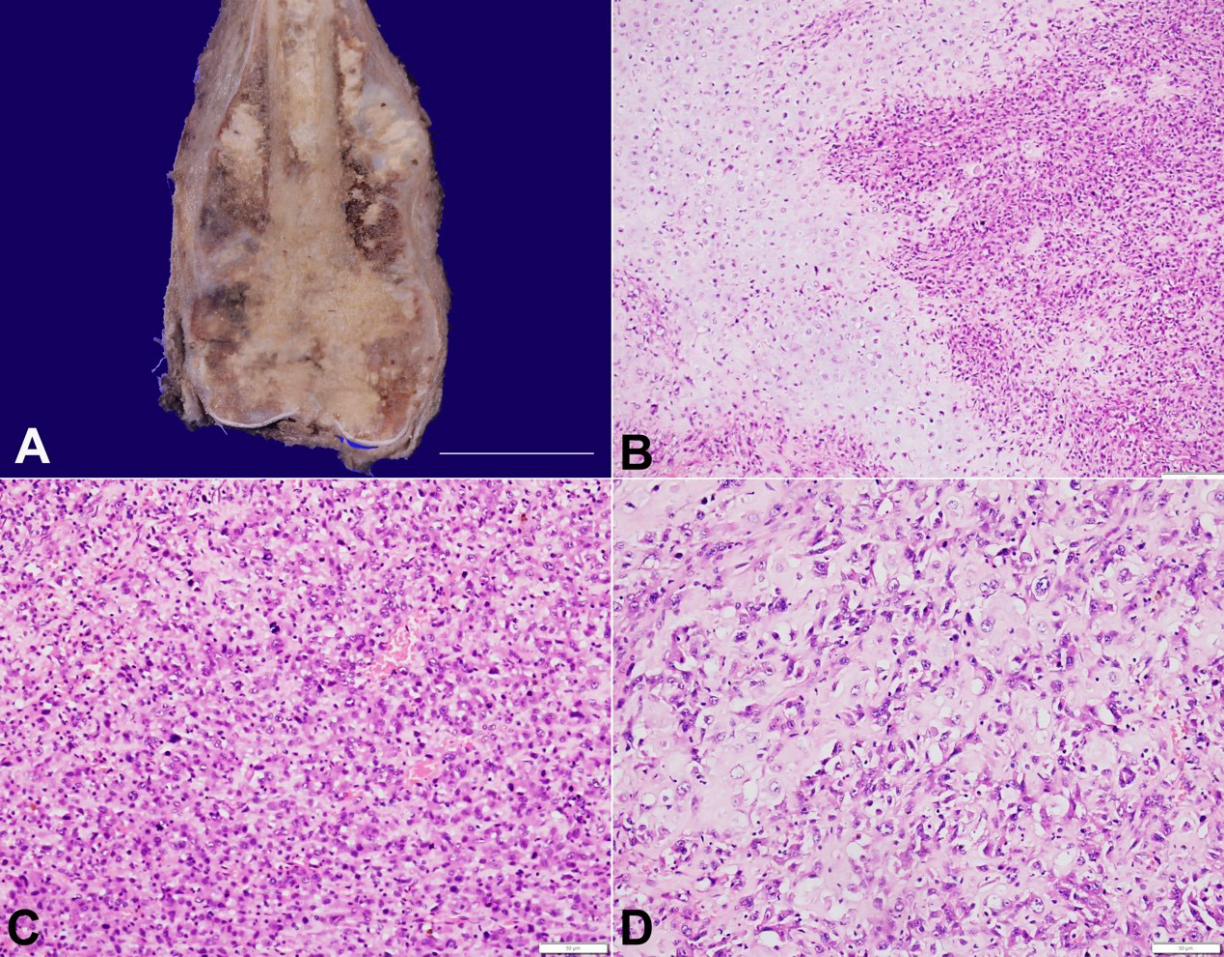
**A –** Gross view of the resected femur showing a grey-white infiltrative tumor involving the lower end of the femur, causing periosteal elevation and intramedullary extension. The mucoid to homogenous white areas represent the cartilaginous islands with intervening calcification (scale bar = 12cm); **B –** Photomicrograph showing permeative cellular tumor (right) condensing at cartilaginous islands (left) (H&E; x100); **C –** Pleomorphic tumor cells with brisk mitosis (H&E; x200); **D –** Malignant lacy osteoid permeating the markedly atypical cartilaginous cells (H&E; x200).

Figure 1 describes a 15-year-old female with rapidly increasing swelling and pain in her left leg. On examination, there was local tenderness and erythema. Computed tomography showed a destructive lytic lesion involving the diaphysis and epiphysis of the lower femoral end with a periosteal reaction. There was soft tissue extension as well, and the patient was given the diagnosis of osteosarcoma. He underwent chemotherapy with poor response to treatment and was referred to our tertiary care center for limb salvage surgery. The excised specimen of the left femur showed an expanded distal condylar end. On bivalving, a grey-white hard tumor measuring 17.5×11.5×8.8cm was seen with variegated areas of hemorrhage, myxoid change, and calcification (Figure 1A). The tumor was seen extending into the medullary cavity up to the neck. The cortex was breached with periosteal reaction and surrounding soft tissue extension. The articular, soft tissue, muscular, and neurovascular margins were tumor-free. On microscopy, there was sheeting, the lobular and fascicular pattern of cellular tumor infiltrating into the bone and soft tissue (Figure 1B). The tumor cells showed moderate to marked pleomorphism with vesicular nuclei, conspicuous nucleoli, and a moderate amount of cytoplasm. Intervening the tumor cells, a significant amount of lacy-like osteoid material deposition was present. Frequent mitosis was seen; however, no necrosis was identified (Figure 1C). There were nests and lobules of cartilage with increased cellularity and a marked degree of nuclear atypia along with osteoid matrix material (Figure 1D). All the resection margins were not involved in the tumor. Based on clinical, radiological, and histopathological findings, the diagnosis of chondroblastic osteosarcoma [pT2; American Joint Committee on Cancer (AJCC) staging manual; 8th edition] was rendered.
